# Engineering of CYP153A33 With Enhanced Ratio of Hydroxylation to Overoxidation Activity in Whole-Cell Biotransformation of Medium-Chain 1-Alkanols

**DOI:** 10.3389/fbioe.2021.817455

**Published:** 2022-01-03

**Authors:** Hyuna Park, Doyeong Bak, Wooyoung Jeon, Minjung Jang, Jung-Oh Ahn, Kwon-Young Choi

**Affiliations:** ^1^ Department of Environmental Engineering, College of Engineering, Ajou University, Suwon, South Korea; ^2^ Department of Environmental and Safety Engineering, College of Engineering, Ajou University, Suwon, South Korea; ^3^ Biotechnology Process Engineering Center, Korea Research Institute of Biosceince and Biotechnology (KRIBB), Cheongju, South Korea; ^4^ Department of Bioprocess Engineering, University of Science and Technology (UST), Daejeon, South Korea

**Keywords:** enzyme engineering, CYP153A33, α,ω-alkanediol, over-oxidation, whole-cell biotransformation

## Abstract

α,ω-Dodecanediol is a versatile material that has been widely used not only as an adhesive and crosslinking reagent, but also as a building block in the pharmaceutical and polymer industries. The biosynthesis of α,ω-dodecanediol from fatty derivatives, such as dodecane and dodecanol, requires an ω-specific hydroxylation step using monooxygenase enzymes. An issue with the whole-cell biotransformation of 1-dodecanol using cytochrome P450 monooxygenase (CYP) with ω-specific hydroxylation activity was the low conversion and production of the over-oxidized product of dodecanoic acid. In this study, CYP153A33 from *Marinobacter aquaeolei* was engineered to obtain higher ω-specific hydroxylation activity through site-directed mutagenesis. The target residue was mutated to increase flux toward α,ω-dodecanediol synthesis, while reducing the generation of the overoxidation product of dodecanoic acid and α,ω-dodecanedioic acid. Among the evaluated variants, CYP153A33 P136A showed a significant increase in 1-dodecanol conversion, i.e., 71.2% (7.12 mM from 10 mM 1-dodecanol), with an increased hydroxylation to over-oxidation activity ratio, i.e., 32.4. Finally, the applicability of this engineered enzyme for ω-specific hydroxylation against several 1-alkanols, i.e., from C6 to C16, was investigated and discussed based on the structure-activity relationship.

## Introduction

Cytochrome P450 monooxygenases (CYPs) are oxidoreductases that catalyze the insertion of an oxygen atom into diverse substrates, with excellent regio-/stereo-selectivity (Park et al., 2020b); CYP consists of heme-thiolate structures in its catalytic core. CYP enzymes are classified into several groups depending on the electron transfer system of redox proteins. In general, bacterial CYPs belong to the class I system, which consists of a CYP core harboring a heme domain, ferredoxin, and ferredoxin reductase, which require independent expression during the oxidation reaction (Finnigan et al., 2020).

Its substrate spectrum includes fatty derivatives such as fatty alkanes, alcohols, and acids (Jung et al., 2016; Hsieh et al., 2018), of which fatty alcohols are a promising feedstock in the chemical and biochemical industries. Several fatty alcohols have been used as repeating monomers in the polymer industry, or as solvents, lubricants, surfactants, and precursors for organic synthesis (Lee et al., 2018). In particular, α,ω-alkanediols are versatile chemicals that can be obtained *via* the consecutive oxidation of alkanes/1-alkanol or the reduction of diacids by carboxylic acid reductase (Kirillova et al., 2009; Olmedo et al., 2016; Hsieh et al., 2018). They are also widely used as monomer precursors for polyesters, polyamides, and polyurethane, through cascade oxidation, amination, and polymerization reactions (Ahsan et al., 2018). For example, α,ω-alkanediols are widely used as building blocks for polyester synthesis through direct esterification reactions (Dai et al., 2017).

During the oxidation of long-chain fatty derivatives, however, regioselectivity is critical for obtaining high-purity products, varying depending on the CYP enzyme family and its carbon chains (Munday et al., 2016); for example, the most well-known CYP102A1, also called BM3, was reported to prefer ω-1 or ω-2 regioselectivity over the ω-position (Whitehouse et al., 2011; Whitehouse et al., 2012; Li and Wong, 2019). In addition, CYP505 and CYP102A showed distinct preferable ratios at the ω-1, ω-2, and ω-3 positions; a CYP enzyme responsible for ω-carbon-specific hydroxylation has also been identified (Whitehouse et al., 2012). The CYP153 family, for example, has been isolated and characterized to function as an ω-specific hydroxylase of long-chain fatty derivatives; additionally, CYP2E1, CYP4A1, and CYP153A G307, all prefer ω-specific hydroxylation on the ω-1, 2, and 3 positions (Holmes et al., 2004; Kuzgun et al., 2020). The CYP153A family is classified as a class I CYP enzyme, requiring redox proteins to mediate electron transfer (Van Beilen et al., 2006). Several CYP153 subfamily members, such as CYP153A7 and CYP153A33, showed a wide range of substrate specificities, such as ω- and ω-1 specific hydroxylation activity against C12 fatty acids, and have demonstrated a high degree of structural plasticity and flexibility in substate recognition sites (Fujita et al., 2009; Fiorentini et al., 2018).

To utilize the CYP153 family for preparing α,ω-alkanediols from alkanes or 1-alkanol, it is necessary to secure the active CYP153 enzyme with high regioselectivity against the targeted chain of substrates. Another limiting factor in such bioconversion is the overoxidation activity of the CYP153 enzyme, which is known to proceed with the continuous oxidation of the produced alcohols into acid *via* aldehyde. Although the oxidation of alcohol and aldehyde reportedly occurs *via* alcohol dehydrogenase or fatty alcohol oxidase and aldehyde dehydrogenase enzymes, respectively, some CYP enzymes have also been reported to catalyze overoxidation reactions as well (Scheller et al., 1998; Eschenfeldt et al., 2003). Although the exact reaction mechanisms of overoxidation are not clearly understood and need clarification, the overoxidation products can be attributed to the catalytic activity of CYP in converting alcohol to aldehyde, followed by oxidation to acids. According to previous reports, purified CYP52A3 from *Candida maltose* could oxidize the first oxidation product of 1-hexadecanol, thus generating 1,16-hexadecanedioic acids without the intervention of other enzymes such as ADH and FAO (Scheller et al., 1998). Similarly, studies demonstrated that CYP52A13 and CYP52A17 isolated from *Candida tropicalis* (ATCC20336) displayed overoxidation activity on long-chain saturated and unsaturated fatty acids; postulating the path of the NADPH-dependent conversion of fatty acids to corresponding fatty aldehydes and α,ω-dicarboxylic acid (Eschenfeldt et al., 2003).

Previously, our group reported the production of 1,12-dodecanediol from 1-dodecanol or dodecane as a substrate, using CYP153A from *Marinobacteri aquaeolei* and Nfa22290 from *Nocardia farcinica* (IFM10152) in combination with putida ferredoxin and ferredoxin reductase (CamA) (Park and Choi, 2020). During the biotransformation of dodecane, the conversion to diol was less than 10%, and its overoxidation products, i.e., dodecanoic acid (lauric acid) and 12-hydroxydodecanoic acid were observed as byproducts. This suggests that overoxidation limited the production of α,ω-alkanediols, and needs to be engineered to obtain CYP153A deficient in overoxidation activity.

Based on our structural understanding, CYP153A enzymes appear to have a high degree of structural plasticity and flexibility in the catalytic core site that hosts the substrate recognition site. Structural analysis of the CYP153A33 enzyme to enhance terminal hydroxylation of fatty acids has also been reported; the substrate-binding pocket of the enzyme has an inverted conical shape. It has thus been suggested that the fatty acid substrate can be vertically combined (Sara et al., 2016). In this study, the activity ratio of ω-site-specific hydroxylation to overoxidation by CYP153A33 variants was increased by mutation of the substrate-binding pocket, i.e., by substituting key amino acids through the destruction of proline-proline linkages. In addition, whole-cell biotransformation by wild-type CYP153A33 and its mutants was investigated with medium- and long-chain fatty alkanols. Interestingly, the CYP153A33 mutant displayed significantly higher ω-specific hydroxylation activity on dodecanoic acid and a low ratio of overoxidation activity, when compared to the wild-type. These results would be of great help for further research on the CYP-dependent oxidation of fatty derivatives.

## Materials and Methods

### Chemical Reagents and Media

All fatty alcohols, fatty acids, and solvents were obtained from Sigma-Aldrich (Seoul, South Korea) and Sejin CI (Seoul, South Korea). The culture media used in this research were obtained from Becton, Dickinson, and Company, U.S., and the ethyl acetate solvent was obtained from Samchun Chemical Co. Ltd., South Korea.

### Site-Directed Mutagenesis of CYP153A33 and Construction of Expression System in *E. coli*

Mutations in the protein structure were induced through site-directed mutagenesis, using the pair of primers, 5′-CAG​CCC​CTC​CGC​AGG​GTC​ACC​GAG-3′, and 5′-CTC​GGT​GAC​CCT​GCG​GAG​GGG​CTG-3′. After the preparation of two PCR templates, the final PCR was conducted using another pair of primers (i.e., 5′-AAA CAT ATG ATG CCA ACA CTG CCC AGA-3′, and 5′-AAA CTC GAG TTA ACT GTT CGG TGT CAG-3′); restriction sites were NdeI and XhoI. The enzyme expression system was identical to that used in previous research (Park et al., 2020b). Putidaredoxin *camB* and putidatedoxin reductase *camA* from *Pseudomonas putida* were overexpressed as the redox proteins for CYP catalysis, and the long-chain fatty acid transporter *fadL* from *Escherichia coli* was overexpressed in *E. coli* BW25113(DE3)Δ*fadD* (Bae et al., 2014; Park and Choi, 2020). The CYP153A33 and mutant genes were each inserted into a pET-24ma (+) expression vector, and the *camA* and *camB* genes were cloned into a pETduet-1 expression vector; the *fadL* gene was cloned into pCDFduet-1 MCS1. The mutant CYP153A33 P136A was also inserted into a pET-24ma (+) expression vector, and was included in an identical whole-cell transformation system (Park and Choi, 2020).

### Enzyme Expression and Whole-Cell Reaction of Primary Fatty Alcohols Using Recombinant *E. coli* Cells

Recombinant *E. coli* BW25113(DE3)Δ*fadD* was cultured in a Luria-Bertani medium containing kanamycin, ampicillin, and spectinomycin at 37°C for 9 h with shaking at 200 rpm (Bae et al., 2014; Park and Choi, 2020). The cells were then grown in 50 ml of Terrific Broth containing the same antibiotics, at 37°C for approximately 4 h until an OD_600_ of 2.4, was reached; Erlenmeyer baffled flasks were used for culturing. Next, 0.5 mM 5-aminolevulinic acid (ALA), 0.25 mM of isopropyl-β-D-thiogalactopyranoside (IPTG), and 0.1 mM iron (II) sulfate were added for protein expression. Induced cells were incubated at 30°C for 10 h with shaking at 200 rpm. For whole-cell reaction, the cells were harvested by centrifugation at 8,000 rpm for 10 min and then washed twice with a 0.1 M potassium phosphate buffer (pH 7.0). The cell pellets thus obtained were resuspended in 0.1 M potassium phosphate buffer containing 1% (w/v) D-glucose and diluted to an OD_600_ of 30. Reactant (10 ml) containing 10 mM of 1-alkanol was incubated at 30°C and 200 rpm using a 100-ml Erlenmeyer baffled flask.

### Gas Chromatography Analysis

Whole-cell reaction samples were analyzed using gas chromatography. Ingredients in the samples were extracted with an equal amount of ethyl acetate at 50°C. The supernatant was separated by centrifugation and derivatized into N,O-bis(trimethylsilyl) trifluoroacetamide at 70°C for 40 min. The derivatized sample was analyzed using a 6500 GC gas chromatography system (Younglin, Suwon, South Korea); an Agilent J&W GC column (CP-Sil 5 CB, 30 m, 0.25 mm i.d.; 0.25 μm film thickness) was used for its analysis. The conditions for the detection of 1-dodecanol, α,ω-dodecanediol, dodecanoic acid, and ω-hydroxydodecanoic acid are mentioned below (Park and Choi, 2020). The initial column temperature was 80°C, which was then increased to 230°C at 20°C/min and maintained for 1.5 min. The capillary flow rate was 2 ml/min, and the carrier gas was nitrogen (N_2_); the same column was used for the detection of other extracts as well. The initial column temperature was 130°C, which was then increased to 230°C at 20°C/min and maintained for 3 min.

### Structural Analysis of Enzyme and Enzyme-Substrate Docking Simulation

Enzyme-substrate docking simulation was conducted using PyMOL2 (Schrödinger, Inc., US). Structural analysis of CYP153A33 was conducted using AutoDockTools-1.5.6 (Scripps Research, San Diego, CA), using PDB 5FYF (DOI**:** 10.2210/pdb5FYF/pdb) as the template.

## Results

### Whole-Cell Transformation of 1-Dodecanol by CYP153A33 Expressing *E. coli*

**FIGURE 1:**
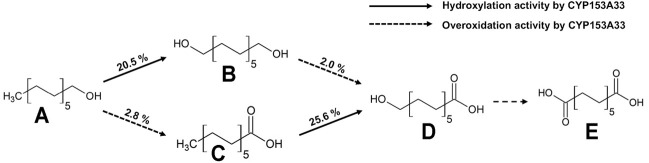
Multistep cascade reaction of 1-dodecanol by CYP153A33-dependent whole-cell transformation. Conversion was calculated when CYP153A33 reached the highest α,ω-dodecanediol production. Conversion of dodecanoic acid to ω-hydroxydodecanoic acid was conducted independently, using dodecanoic acid as a substrate; **(A)** 1-dodecanol, **(B)** α,ω-dodecanediol, **(C)** dodecanoic acid, **(D)** ω-hydroxydodecanoic acid, **(E)** dodecanedioic acid.

The CYP153A33-encoding gene was co-expressed with CamAB redox proteins for the whole-cell biotransformation of 1-dodecanol. 10 mM of 1-Dodecanol (A) was used as the substrate, and the whole-cell bioconversion resulted in diverse production profiles, including α,ω-dodecanediol (B), dodecanoic acid (C), ω-hydroxydodecanoic acid (D), and α,ω-dodecanedioic acid (E) (Figure 1). After 3 h of reaction, the production titer of α,ω-dodecanediol was the highest, and each product was separated by gas chromatography for simultaneous quantitative analysis. The production profile with conversion included 20.5% α,ω-dodecanediol, 2.8% dodecanoic acid, 2.0% ω-hydroxydodecanoic acid, plus miscible amounts of α,ω-dodecanedioic acid. The conversion of ω-hydroxydodecanoic acids to dodecanedioic acid was also observed as less than 5%, indicating that ω-specific hydroxylation to the terminal carbon of the fatty substrate is the most dominant reaction. The relatively lower dodecanoic acid accumulation might be due to the reduction reaction of the aldehyde to alcohol by endogenous enzymes in the host strain of *E. coli* BW25113(DE3)Δ*fadD*.

The major product was identified as α,ω-dodecanediol, but the conversion was low, i.e., only 23.3%. Although one of the overoxidation products of 1-dodecanal was not detected in the whole-cell biotransformation, another overoxidation product of ω-hydroxydodecanoic acid was generated with a similar amount of dodecanoic acid, whose hydroxylation ratio to overoxidation activity was calculated as 13.7. In a control experiment that converted 1-dodecanol to dodecanoic acid by using *E. coli* BW25113(DE3)Δ*fadD* cells harboring an empty vector, less than 0.1 mM of dodecanoic acid was produced, suggesting that this conversion was attributed to both CYP153A33 and the endogenous oxidase enzymes in host cells. In addition, a separate experiment for dodecanoic acid bioconversion *via* CYP153A33-expressing cells resulted in 25.6% conversion to ω-hydroxydodecanoic acid, while 2% conversion was observed in the 1-dodecanol bioconversion. This suggests that CYP153A33 favors fatty acid forms as a substrate, rather than 1-alkanol and overoxidation activity, which was higher with α,ω-dodecanediol (9.8%) than it was with 1-dodecanol (2.8%).

### Overoxidation Activity of CYP153A33 and Sequence Analysis With CYP52A Family

Understanding the correlation between end-point production and rate at each step revealed that the highest rate could be observed in the 1-dodecanol conversion, while the lowest was observed in the ω-hydroxydodecanoic acid conversion. These oxidation patterns are in accordance with previous results by Scheller et al., wherein the purified CYP52A3 displayed overoxidation activity during the oxidation of hexadecane, which was further confirmed using kinetic studies demonstrating higher V_max_ values against alkane, 1-alkanol, 1-alkanal, and lower V_max_ against acid, diol, and hydroxy acid.

CYP153A33 showed slight homology (around 20%) with CYP52A3, CYP52A13, and CYP52A17, which were all previously proven to have good overoxidation activity; however, some conserved regions were found in the substrate-binding domains and the active site. Firstly, the residues at the entrance of the substrate access channel may differentiate in their substrate specificity and binding affinity with the -OH functional group of the hydroxylated product. Secondly, the CYP153A33 and CYP52A families contain a conserved sequence (i.e., NXXLLXIVXGXDTT) in the central I-helix, suggesting that these residues might be responsible for the high ω-regioselectivity of the CYP153A33 and CYP52A families. One important clue about this overoxidation activity appears to lie in the feedback regulation, as the final product (i.e., α,ω-dodecanedioic acid) acts as a competitive inhibitor of 1-dodecanol binding and may be important for the metabolic regulation of P450 activity (Scheller et al., 1998).

### Selection of Key Residue in Substrate Binding Pocket and Site-Directed Mutagenesis of CYP153A33

**FIGURE 2:**
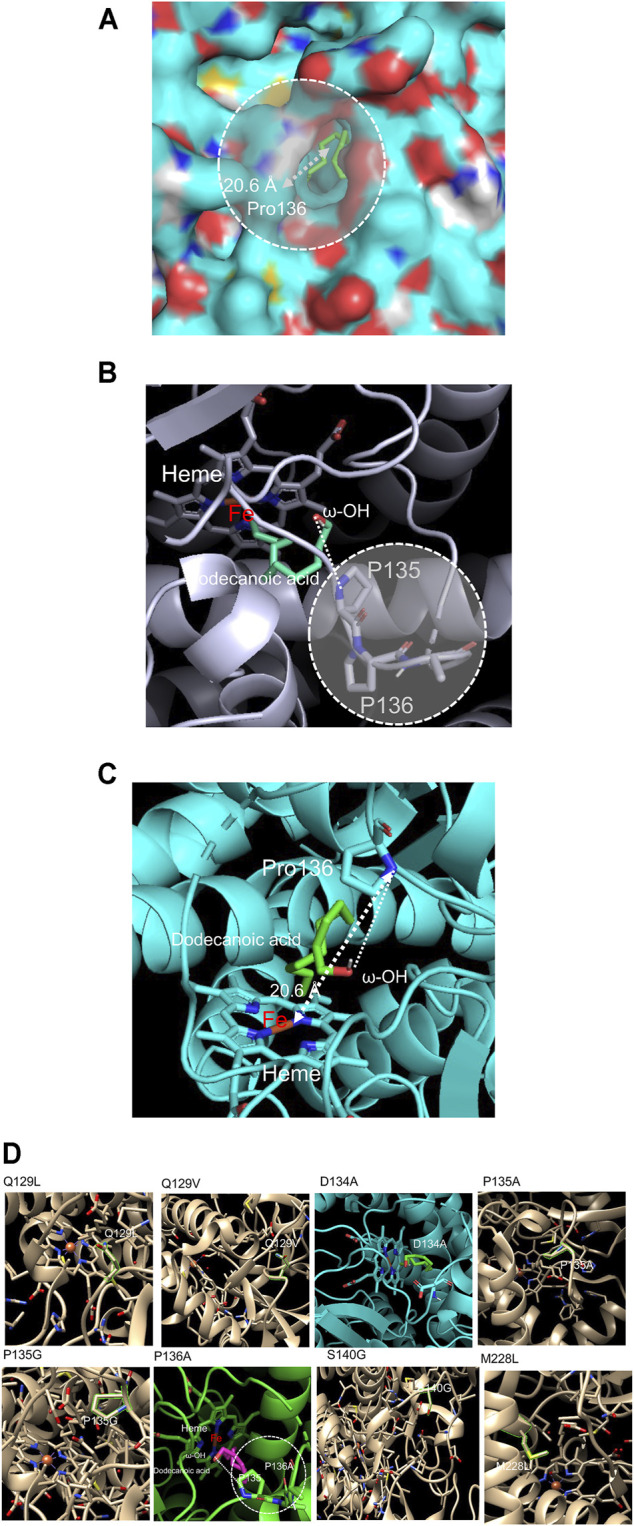
**(A)** Construction of a CYP153A33 structure model, used to calculate the distance between the heme center and hydroxyl functional group of docked dodecanoic acid. **(B)** 1-dodecanol-docked CYP153A33 structural model; P135-P136 linkage was found at the substate recognition site which holds ω-OH terminal of the docked dodecanoic acid. **(C)** Among two proline residues, Pro136 has direct interaction with the ω-OH terminal of docked dodecanoic acid. **(D)** Selection of target residues in the active site and substrate binding site of CYP153A33. Among the screened residues, D134A and P136A were finally selected for the evaluation of 1-dodecanol bioconversion.

Based on the analysis of conserved sequences of the CYP153A33 and CYP53A families, we attempted to engineer CYP153A to lower the overoxidation activity by mutating key residues binding the generated hydroxyl functional group. Firstly, a structural model of CYP153A33 was constructed based on the crystal structure of CYP153A (PDB code: 5FYF), followed by docking simulation with a dodecanoic acid substrate. The constructed model showed an obvious path from the hydroxyl functional group of dodecanoic acid to the heme active site, allowing the long-chain fatty acids to access and bind to one another without hindrances. Additionally, the distance of the linear path was calculated to be 20.6 Å (Figure 2A).

On the active site in the pocket of the dodecanoic acid-docked CYP153A33 model, proline-proline contiguous amino acid sequences at Pro135 and Pro136 were identified (Figure 2B). Proline residues are known to act as structural disruptors in the middle of regular secondary structure elements such as α-helices and β-sheets, thereby increasing the rigidity of protein structures and inhibiting the flexibility of enzymes when incorporated into peptide bonds in the active site (Morgan and Rubenstein, 2013). Since Pro135 and Pro136 were located in the heme active site, bound to the expected hydroxylated carbon atom of α,ω-dodecanediol to facilitate further oxidation to aldehyde by holding and facing the substrate-enzyme complex to the heme active site, Pro136 in the proline-proline linkage was selected as the first target for a mutation (Figure 2C). In addition, key residues including Gln129, Asp134, Met129, and Ser140 in the substrate recognition site were identified, and their mutation was first investigated via docking simulation with 1-dodecanol. The selected residues were exchanged with smaller, hydrophobic residues to minimize the effect on pocket polarity and to increase the chance of substrate access to the active site by lowering the rigidity of the substrate access path. Mutated CYP153A33 structures—Q129L, Q129V, D134A, P135A, P135G, P136A, S140G, and M228L—were generated and evaluated (Figure 2D). Excluding D134A and P136A, the docking model did not show a dramatic decrease in the affinity energy and distance from the heme iron center. Next, CYP153A33 D134A and P136A mutants were constructed and their whole-cell activities were investigated.

### Whole-Cell Transformation of 1-Dodecanol by CYP153A33 D134A and P136A

**FIGURE 3:**
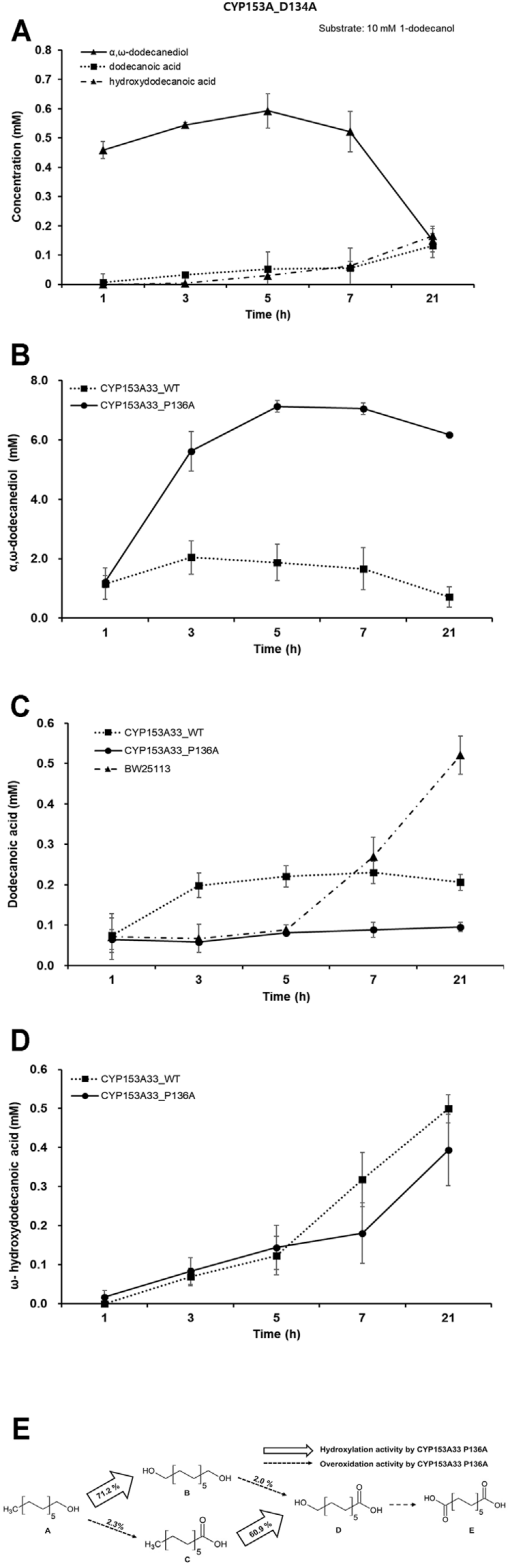
Evaluation of 1-dodecanol bioconversion by whole-cell reaction of CYP153A33 mutant strains. **(A)** Whole-cell biotransformation of 1-dodecanol by CYP153A33 D134A. The production time-profile includes α,ω-dodecanediol, dodecanoic acid, and ω-hydroxydodecanoic acid, from 10 mM of 1-dodecanol substrate. **(B)** Production of α,ω-dodecanediol, **(C)** dodecanoic acid, and **(D)** ω-hydroxydodecanoic acid from 10 mM of 1-dodecanol substrate, through whole-cell biotransformation of 1-dodecanol using CYP153A33 P136A (solid line) and wild-type (dotted line) strains. **(E)** Multistep cascade reaction of 1-dodecanol by CYP153A33 P136A-dependent whole-cell transformation. Conversion was calculated when CYP153A33 P136A reached the highest α,ω-dodecanediol production. Conversion of dodecanoic acid to ω-hydroxydodecanoic acid was conducted independently, using dodecanoic acid as a substrate; **(A)** 1-dodecanol, **(B)** α,ω-dodecanediol, **(C)** dodecanoic acid, **(D)** ω-hydroxydodecanoic acid, **(E)** dodecanedioic acid.

Whole-cell biotransformation of 10 mM 1-dodecanol was performed with a recombinant *E. coli* system expressing each CYP153A33 variant of D134A and P136A; however, no significant increase in bioconversion activity was observed in the mutant strains of D134A. The highest tier could be reached after 5 h of reaction, and less than 0.7 mM of α, ω-dodecanediol was produced from 10 mM 1-dodecanol, which was much less than that of wild-type CYP153A33 (Figure 3A). Other oxidative metabolites of dodecanoic acid and ω-hydroxydodecanoic acid were produced less than 2% conversion.

Interestingly, among the mutant strains, only the CYP153A33 P136A mutant showed a significant increase in α,ω-dodecanediol production, while also having a dramatic decrease in the overoxidation ratio. The conversion of 1-dodecanol to α,ω-dodecanediol was increased up to 71.2% within 5 h of the whole-cell reaction, and it was 3.47 times higher than that of the wild-type CYP153A33 (Figure 3B). Therefore, the hydroxylation ratio over overoxidation activity dramatically increased to 32.4, which was a 2.3-folds increase compared to that of the CYP153A33 wild type. In addition, the conversion of dodecanoic acid to ω-hydroxydodecanoic acid by the P136A mutant independently increased up to 2.38 times with 60.9% conversion. The final overoxidation production of α,ω-dodecanedioic acid was not completely converted by P136A; direct conversion to dodecanoic acid was less than 3% by both CYP153A33 and P136A (Figure 3C). This seems to be because 1-dodecanol was mostly consumed by the P136A mutant for hydroxylation and could be due to the increased amount of conversion to α,ω-dodecanediol, by increasing the access opportunity to the active site of 1-dodeacanol results from the flexibility of the pocket. Similarly, dodecanoic acid accumulated at higher concentrations after 5 h, through wild-type CYP153A33 and *E. coli* control strains not harboring any CYP-redox plasmids than P136A (Figure 3D).

The overall conversion values of CYP153A33 P136A, when the highest conversion of α,ω-dodecanediol production reached, are summarized in Figure 3E for easy comparison with those of wild-type CYP153A33. It is worth noting that the hydroxylation activity against 1-dodecanol and dodecanoic acid was significantly increased and the hydroxylation ratio with overoxidation activity also increased significantly.

### Evaluation of Substrate Specificity of CYP153A33 P136A Against Medium and Long-Chain Fatty Alkanols

**FIGURE 4:**
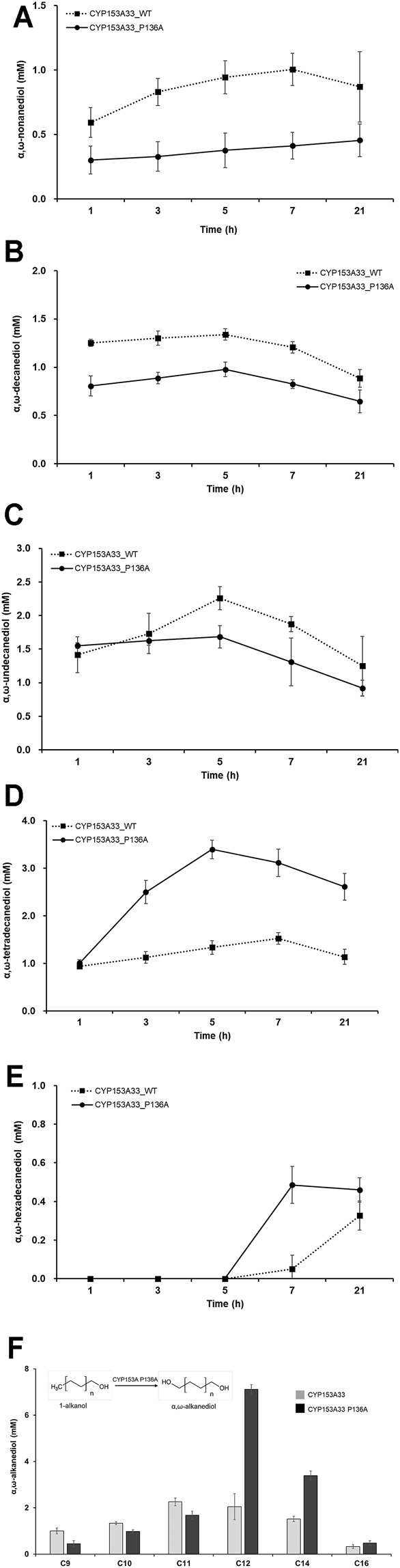
Whole-cell biotransformation of medium- and long-chain 1-alkanols into corresponding α,ω-alkanediols. Time-dependent production profile of α,ω-alkanediol in bioconversion of: **(A)** 1-nonanol, **(B)** 1-decanol, **(C)** 1-undecanol, **(D)** 1-tetradecanol, and **(E)** 1-hexadecanol, using CYP153A33 P136A (solid line) and wild-type (dotted line) strains. **(F)** Summary and comparison of α,ω-alkanediol production using CYP153A33 P136A (black bar) and wild-type (grey bar) strains.

The bioconversion activity of CYP153A33 P136A against medium-and long-chain 1-alkanols was then evaluated. Under similar reaction conditions, the whole-cell reaction was conducted in the presence of 1-alkanol substrates including 1-hexanol (C6), 1-nonanol (C9), 1-decanol (C10), 1-undecanol (C11), 1-tetradecanol (C14), and 1-hexadecanol (C16). The production of each α,ω-alkanediol was first evaluated and compared with the bioconversion activity of the wild-type CYP153A33 (Figures 4A–E). No oxidation product could be found in the C6 bioconversion; CYP153A33 showed higher α,ω-alkanediol production with C9, C10, and C11 1-alkanol, while P136A showed higher conversion with C12, C14, and C16 1-alkanol. In particular, the maximum yield was more than double that of the wild-type, and 3.4 mM of α,ω-tetradecanediol can be produced via 1-tetradecanol bioconversion within 5 h of reaction. In most cases, however, bioconversion was much lower than C12 of 1-dodecanol, suggesting that CYP153A33 P136A accepts C12 fatty alcohol as the most favorable substrate with exceptionally high conversion (Figure 4F), unlike for C14 and C16.

**FIGURE 5:**
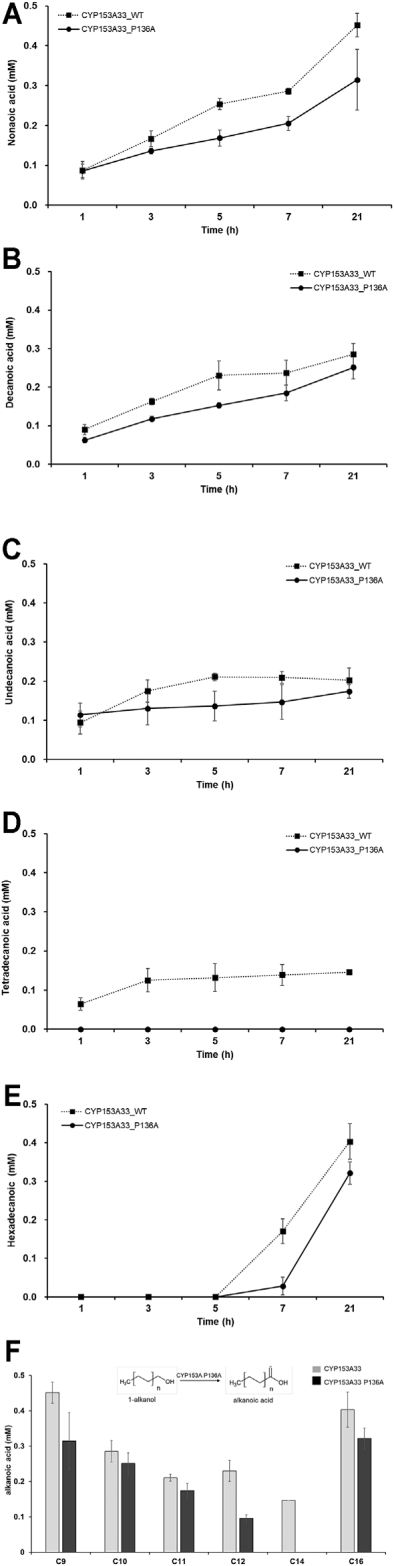
Whole-cell biotransformation of medium- and long-chain 1-alkanols into corresponding fatty acids through overoxidation. Time-dependent production profile of fatty acids in bioconversion of: **(A)** 1-nonanol, **(B)** 1-decanol, **(C)** 1-undecanol, **(D)** 1-tetradecanol, and **(E)** 1-hexadecanol, using CYP153A33 P136A (dotted line) and wild-type (solid line) strains. **(F)** Summary and comparison of fatty acids production using CYP153A33 P136A (black bar) and wild-type (grey bar) strains.

The overoxidation activity of CYP153A P136A with C9 to C16 1-alkanols was also investigated (Figures 5A–E). The lowest overoxidation activity was observed for the P136A mutant against tetradecanoic acid, while the highest was observed for CYP153A33 against nonanoic acid. In general, the P136A mutant displayed lower overoxidation activity throughout the examined (C9 to C16) 1-alkanols compared to the CYP153A33 wild-type. Interestingly, the overoxidation activity of both wild-type CYP153A33 and the P136A mutants decreased as the carbon chain increased from C9 to C12 and increased as the carbon chain increased from C14 to C16, suggesting that the lowest activity is between the C12 and C14 chain-lengths of 1-alkanol. In the 1-tetradecanol bioconversion, no overoxidation product(s) of tetradecanoic acid were observed.

## Discussion

In this study, CYP153A33, a well-known enzyme with ω-specific hydroxylation activity in fatty primary alcohols, was engineered to obtain a higher hydroxylation activity with 1-dodecanol, along with increased α,ω-dodecanediol conversion. As a result, the best variant of CYP153A33 P136A could be selected, and the bioconversion of 1-dodecnaol increased significantly. The overoxidation product decreased significantly or was not observed in 1-alkanol bioconversion by CYP153A P136A, suggesting that the ratio of hydroxylation to overoxidation activity is also critical if it is mediated by endogenous enzymes. One thing that should be addressed here is that the origin of CYP153A33 is bacterial, i.e., it is not from a yeast host. Since overoxidation activity was observed by the CYP52A family from *Candida* species of yeast, the catalytic activity and overoxidation-related mechanisms could differ (Scheller et al., 1998; Eschenfeldt et al., 2003). The sequence identity between the CYP52A family and CYP153A33 was less than 20%, and both have different electron transfer systems. Although this study targeted the production of α,ω-alkanediol, the overoxidation product of α,ω-alaknedioic acid is also a very useful biochemical for the chemical industry, potentially being used as polymer building blocks, surfactants, and lubricants. Therefore, if extensive, overoxidation could be beneficial for such purposes, by providing a direct route to the α,ω-alkanedioic acid.

In terms of the rates of conversion and production, conversion of more than 70% within 3 h of the whole-cell reaction is very promising, especially in bioprocesses that include CYP bioconversion. In addition, most CYP-dependent oxyfunctionalizations have low turnover rates and NAD(P)H cofactor utilization. The engineered CYP153A33 P136A strain can produce α,ω-dodecanediol with a productivity of 0.29 g/L/h. This value is very promising, considering the reported space-time yields of CYP-dependent whole-cell biotransformation (Park et al., 2020a).

The application of overoxidation deficient CYP enzymes for alkane oxidation is very diverse. For example, direct use of alkanediol for various monomers for polyesters, polyamides, and polyurethane can be possible. Also, preparation of diamine, which can be applied for polyamide monomers, by introducing an amine functional group through cascade oxidation and transamination can be possible. This diamine synthesis is very competitive compared to the method by fatty acid decarboxylase enzymes (Cha et al., 2021). However, there are still more rooms to be engineered for the more efficient whole-cell biotransformation of the CYP153A P136A. For example, engineering approach could be made available by providing additional redox potentials or by introducing transporting channels into the host cell membrane (Choi et al., 2014; Park and Choi, 2020); this would contribute to a higher production titer, as previously reported. A limitation of the CYP-dependent oxidation process is the use and additional and required feeding of aminolevulinic acid as a heme precursor to activate the CYP core structure (Kim et al., 2017; Namgung et al., 2019). This heme precursor is expensive and must be fed extracellularly during induction. Also, the supply of oxygen, which is one of the co-substrates in CYP-dependent oxidation reaction, could be one of the limiting factors. Altogether, there are still challenges to be overcome and solutions to be potentially engineered in further research.

## Data Availability

The original contributions presented in the study are included in the article/Supplementary Material, further inquiries can be directed to the corresponding authors.
